# Involvement of IKAP in Peripheral Target Innervation and in Specific JNK and NGF Signaling in Developing PNS Neurons

**DOI:** 10.1371/journal.pone.0113428

**Published:** 2014-11-19

**Authors:** Anastasia Abashidze, Veronica Gold, Yaron Anavi, Hayit Greenspan, Miguel Weil

**Affiliations:** 1 Laboratory for Neurodegenerative Diseases and Personalized Medicine, Department of Cell Research and Immunology, The Sagol School of Neurosciences, The George S. Wise Faculty of Life Sciences, Tel Aviv University, Tel Aviv, Israel; 2 Department of Applied Mathematics, School of Mathematical Sciences, Tel Aviv University, Tel Aviv, Israel; 3 Department of Biomedical Engineering, Faculty of Engineering, Tel Aviv University, Tel Aviv, Israel; Stanford University School of Medicine, United States of America

## Abstract

A splicing mutation in the *ikbkap* gene causes Familial Dysautonomia (FD), affecting the IKAP protein expression levels and proper development and function of the peripheral nervous system (PNS). Here we attempted to elucidate the role of IKAP in PNS development in the chick embryo and found that IKAP is required for proper axonal outgrowth, branching, and peripheral target innervation. Moreover, we demonstrate that IKAP colocalizes with activated JNK (pJNK), dynein, and β-tubulin at the axon terminals of dorsal root ganglia (DRG) neurons, and may be involved in transport of specific target derived signals required for transcription of JNK and NGF responsive genes in the nucleus. These results suggest the novel role of IKAP in neuronal transport and specific signaling mediated transcription, and provide, for the first time, the basis for a molecular mechanism behind the FD phenotype.

## Introduction

Familial Dysautonomia (FD) is an autosomal recessive neurodegenerative disease, characterized by abnormal development and function of the sensory and autonomic nervous systems [1], [2]. 99.5% of all FD cases show a mutation in the donor splice site of intron 20 of the *ikbkap* gene (IVS20+6T to C). This mutation causes tissue specific skipping of exon 20 and a premature open reading frame termination of the IKAP protein. Of several tissues examined, the central and peripheral nervous systems express the lowest levels of the wild type *ikbkap* mRNA and these are also the tissues most affected in FD [3], [4], [5]. IKAP is a well-conserved 150-kDa protein, which was discovered as a scaffold protein in the IkB kinase (IKK) complex [6] and relates to ELP1/IKA1 family. *Ikbkap* genes of human, mouse, and chick encode the 1332–1334 amino acid IKAP protein sharing amino acid similarity of 81% and 67% for the mouse and chick respectively with the human homologue. In the nucleus, IKAP is described as the human elongator protein 1 (hELP1), a scaffold protein of the RNA-polymerase-II-mediated transcription elongation complex [7], [8]. However, the majority of IKAP can be found in the cytosol, where it is known to be involved in a number of activities ranging from Jun N-terminal kinase (JNK)-mediated stress signaling in human fibroblasts to regulation of exocytosis and transfer RNA modification in yeast [9], [10]. In addition, recent findings demonstrate IKAP involvement in α-tubulin acetylation, migration, and branching of rat cortical neurons [11].

Although the knockout of *ikbkap* in mice is embryonic lethal [12], creation of a conditional *ikbkap* transgenic mouse revealed the phenotype that recapitulates the major FD phenotypic and neuropathological features [13]. Dorsal root ganglia (DRG) neuronal numbers in *ikbkap* mutant embryos are reduced at perinatal E18.5 and gradually decrease to 10 months of age, while even a slight increase in IKAP levels is enough to ameliorate the phenotype and increase the life span. It is well established that all components of the PNS in vertebrates stem from transient population of the neural crest cells (NCC) [14], which migrate from the neural tube along the dorsoventral pathway and produce sensory neurons of the DRG, the sympathetic and enteric neurons of the autonomic lineage. Shortly after colonization of the primary DRG, NCC differentiate to appropriate sensory subtypes [15]. Meanwhile, axonal outgrowth is initiated to establish proper connections in modality-specific fields in the spinal cord, and in peripheral targets. A recent study by George and colleagues [16] provides analysis of the cellular events that can go awry during sensory neurogenesis *in* a conditional *ikbkap* knockout mouse model. In line with previous observations in chick embryos from Hunnicutt and colleagues [17], it is shown that *IKAP* depletion does not affect NCC migration, pathfinding, or DRG and sympathetic ganglia (SG) formation. Instead, *IKAP* appears to be essential for the second wave of neurogenesis of TrkA-positive nociceptors and thermoreceptors in the DRG. Yet, despite these recent advances in FD research using mouse and chick models, the specific IKAP functions and molecular interactions in the developing neuron, as well as the origin of FD phenotype remain unclear. Here we attempted to elucidate the IKAP role during PNS development in the chick embryo and found that IKAP is required for proper axonal outgrowth and target innervation. We demonstrate *in vivo* and *in vitro* that downregulation of *ikbkap* expression by siRNA interference at these stages has a phenotypic impact on neurite outgrowth, branching and guidance. At the subcellular level, *ikbkap* downregulation in cultured DRG neurons resulted in abnormal growth cone morphology due to an effect on microtubules organization and an aberrant colocalization of IKAP with dynein and pJNK at the axon terminals. Concurrently, a specific impairment in pJNK and NGF dependent transcription was detected in these cells, supporting IKAP involvement in axonal transport and specific signaling mediated transcription in PNS neurons.

## Results

### IKAP is expressed in growing axons in the developing PNS

**Figure 1:**
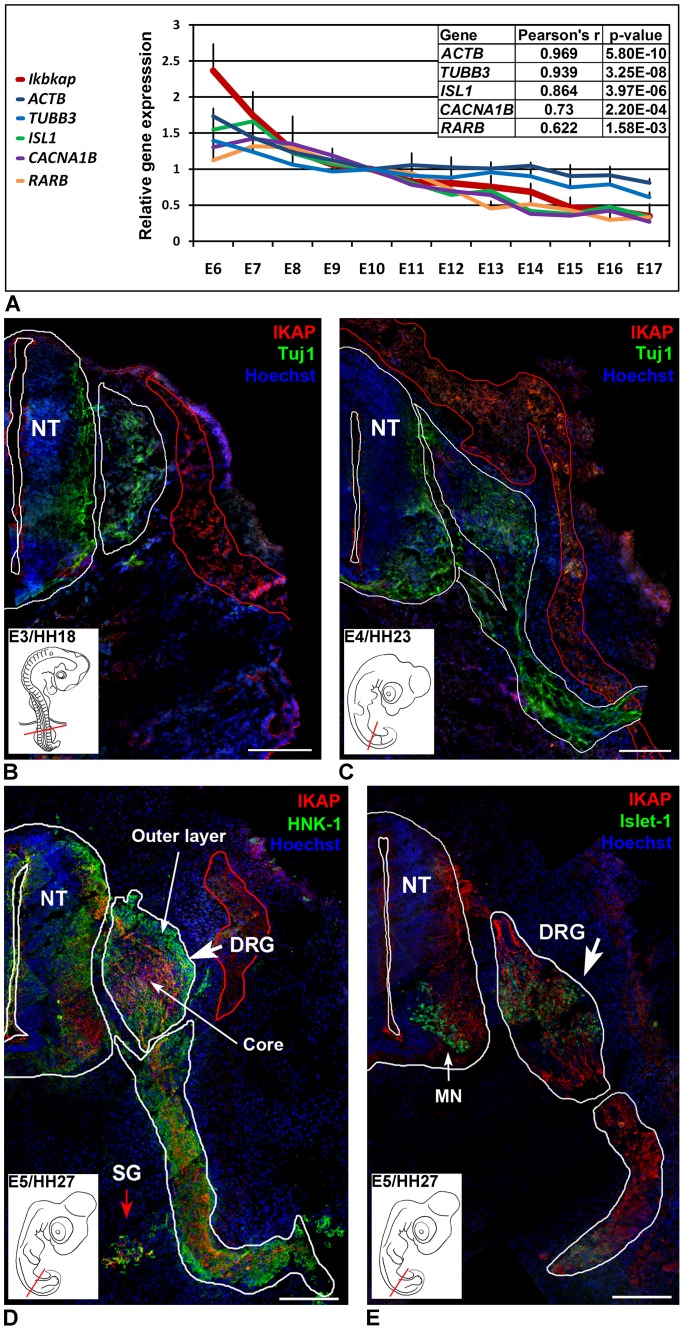
IKAP expression in the developing PNS. (**A**) QRT-PCR analysis of expression levels of *ikbkap*, neural specific βIII-Tubulin (*TUBB3*), β-Actin *(ACTB),* Islet-1 *(ISL1*), Retinoic Acid receptor β (*RARB*), *and CACNA1B* in developing lumbar DRG of E6-E18 embryos. E10 was used arbitrarily as normalizing time point reference. Data from three independent experiments are presented as means ±SD, N = 5 embryos at each time point. Correlation data was obtained following Pearson's correlation test. (**B–E**) Tiling reconstruction showing IKAP expression in transverse sections of E3/HH18-E5/HH27 embryos at the hind limb level, stained with Hoechst 33342 to visualize nuclei (blue), and IKAP specific antibody (red) in combination with Tuj1 (B–C, green), HNK-1 (D, green), or with Islet-1 (E, green). Size bars 100 µm. NT- Neural tube, DRG – Dorsal Root Ganglia, SG – Sympathetic Ganglia, MN – motor neurons.

To evaluate *ikbkap* expression levels in the developing DRG in the context of PNS development, we studied the *ikbkap* expression together with other relevant genes in DRG of E6 to E18 embryos at the lumbar L4, L5, and sacral S1 levels. Quantitative real time PCR (QRT-PCR) analysis shows that *ikbkap* mRNA expression is at the highest level at E6, at the time of intensive neurite outgrowth and peripheral organ innervation (Fig. 1A). In this context, we compared the pattern of *ikbkap* expression to the neural specific βIII-Tubulin (*TUBB3*) and β-Actin (*ACTB*) genes, both are known key players in axonal outgrowth process [18]. Together with this, we related *ikbkap* expression to the homeodomain transcription factor *Islet-1* (*ISL1*), one of the key regulators of sensory differentiation [19]. In this analysis, we found that by Pearson's correlation, *ikbkap* transcript levels are best correlated with the levels of *ACTB* (r = 0.97) and *TUBB3* (r = 0.94), as well as with *ISL1* (r = 0.86). In contrast, *ikbkap* expression is weakly correlated with a promoter of neural progenitor development Retinoic Acid Receptor β (*RARB)* (r<0.62), which was shown to induce neurite outgrowth in NGF-dependent DRG neurons [20]. A weak correlation with *ikbkap* expression (r<0.73) was found for the calcium channel *CACNA1B* gene, which is a representative for the primary calcium release electrophysiological activity that should appear at these early stages of PNS development [21]. Altogether, these results suggest that in the course of DRG development at the time of neuronal innervation *ikbkap* is expressed in a similar temporal pattern with cytoskeletal genes like actin and tubulin, and with a key regulator of sensory differentiation Islet-1.

**Figure 2:**
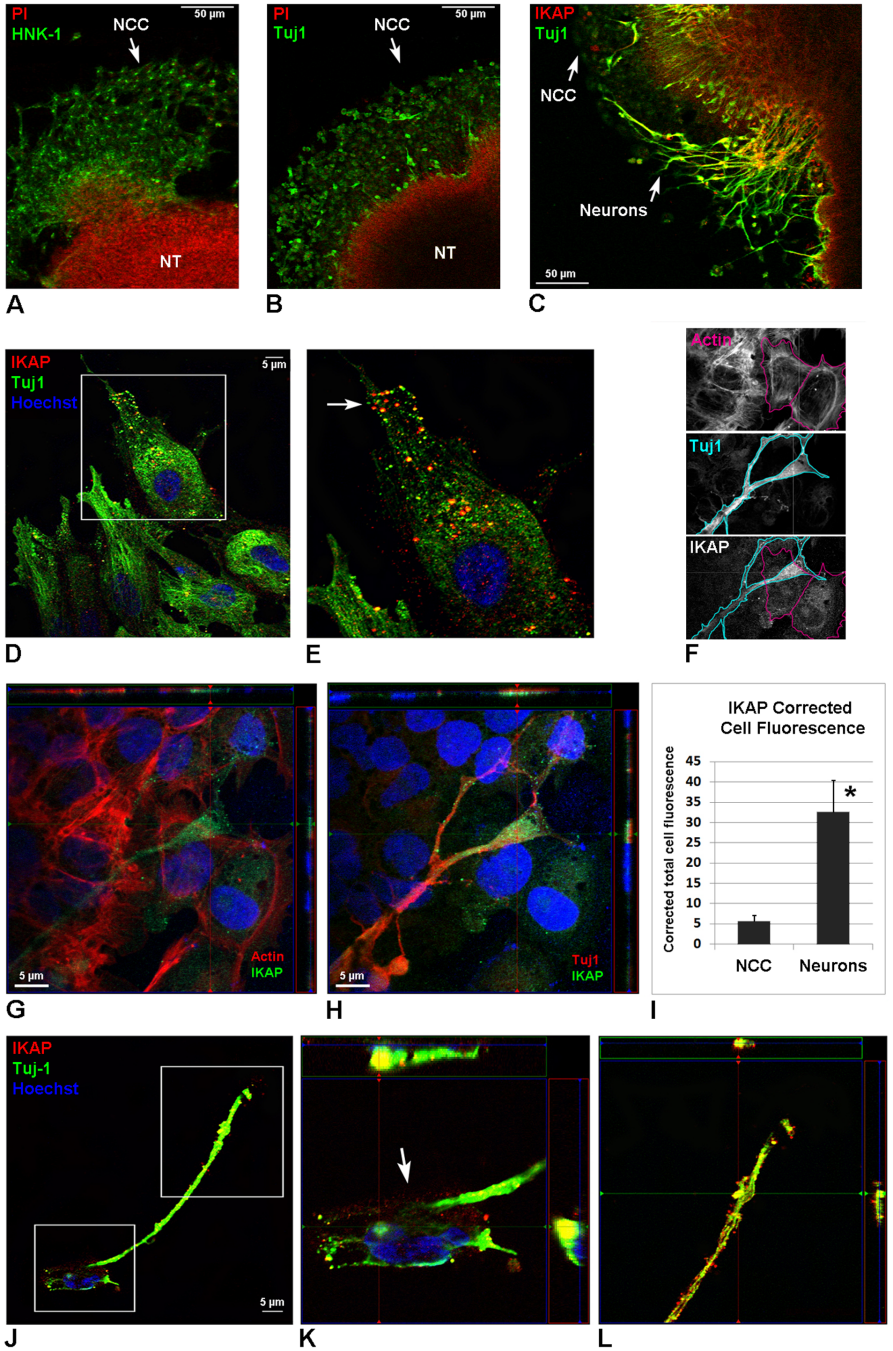
Characterization of IKAP expression in differentiating NCC neuronal cultures. Neural tubes were explanted from the embryos just before the onset of NCC migration (E2/HH11), allowing NCC to migrate and differentiate in culture. (**A–B**) Migrating NCC after 24 h in culture stained for Propidium iodide (PI) to visualize nuclei, specific NCC marker HNK-1 (A), neural specific tubulin (Tuj1, B). **(C)** Migrating NCC after 72 hours in culture stained Tuj1 and PI. (**D–E**) IKAP expression in migrating NCC (24 h in culture) show vesicular pattern and colocalized with Tuj1 at the leading edge of the cell (E, white arrow). Boxed area in D is magnified in E. (**F–H**) Immunofluorescence confocal images of quadruple stained cells show IKAP staining with Alexa-488 conjugated secondary antibody (green in G and H) either in combination with Phalloidin conjugated with TRITC for Actin labeling (red in G) or together with Tuj1 stained with Alexa 647 conjugated secondary antibody (red in H). Hoechst 33342 (blue) was added to stain nuclei in these images. (**F**) For IKAP fluorescence quantification in NCC versus developing neurons (I), NCC cell borders were selected using actin staining (upper panel), neuron cell borders were selected using Tuj1 staining (middle panel), and these cell borders were overlaid on IKAP stained picture (bottom panel). Then Area, Integrated Density, and Mean Gray Value were measured in the cells using ImageJ, and Corrected Total Cell Fluorescence (CTCF) was calculated as described in methods from a sample number of 15 neurons versus 15 NCC. (**G–H**) Two images of differentiating NCC showing the same confocal plane (72 h in culture). Note that IKAP levels increase in the outgrowing neurites of differentiating neurons, and that IKAP is colocalized with Tuj1 (H, red), but not with actin (G, red). (**I**). Corrected Total Cell Fluorescence (CTCF) in NCC and differentiating neurons. Data are represented as mean ±SD. (**J–L**) IKAP expression in NCC derived neurons show vesicular pattern and predominantly is localized along Tuj1 positive extending filaments within growing neurites (L), and to lesser extent at the soma region of the cell (K, indicated by an arrow). Boxed areas in J are magnified in K and L. (G–H) and (K–L) show a set of orthogonal slices, where the middle panel represent the xy plane, left panel represent the yz plane, and upper panel represent the xz plane.

At the protein level, IKAP is absent at E3 and E4 (Fig. 1B–C) but becomes apparent at E5 in the developing DRG neurons and in primary SG (Fig. 1D–E). At E5, the DRG is formed by a core of postmitotic differentiating sensory neurons, which send growing axons toward the spinal cord and to the peripheral targets, and of an outer layer of proliferating neural progenitors. As shown in Fig. 1D, IKAP (labeled in red) is abundantly expressed in growing neurites in the inner core of the DRG, but not in proliferating neural progenitors expressing the neural crest marker HNK-1 (labeled in green) in the outer layer. Confirming IKAP specific expression in postmitotic neurons, we show that IKAP is co-expressed with Islet-1 postmitotic transcription factor [22], (Fig. 1E). In the spinal cord, IKAP is detected in interneurons and motor neurons (MN) expressing Islet-1, and at the entry sites of growing DRG axons dorsally. Interestingly, IKAP is not expressed along the dorsoventral route in early forming PNS organs at E3 and E4 (Fig. 1B and 1C respectively), where the nascent neurons are stained with βIII-type neuron specific tubulin (Tuj1, green). Instead, IKAP is weakly expressed along the dorsolateral NCC migration pathway in distinct population of cells like melanocytes (outlined in red). We also investigated IKAP expression levels during the course of NCC migration and differentiation into neurons *in vitro* (Fig. 2). Neural tubes were explanted from the embryos just before the onset of NCC migration (E2/HH11), allowing the NCC to migrate (Fig. 2A–B) and to differentiate (Fig. 2C) in culture for the period of 24 and 72 hours. After 24 hours in culture, confocal images of NCC show low endogenous IKAP expression, partly co-localized with Tuj-1 (Fig. 2D–E). After 72 hours in culture, IKAP seems to be colocalized with Tuj-1 within the outgrowing neurites (Fig. 2C and 2H–L), but not with actin (Fig. 2G). Moreover, IKAP fluorescence intensity levels increase in the developing neurons at least 6 times compared to migrating NCC as shown by corrected total cell fluorescence (CTCF) quantification in Fig. 2I (explained in methods). Fig. 2J–L show IKAP intracellular localization in an extending neuron. Here IKAP shows a clear vesicular pattern and is distributed along Tuj-1 labeled extending filaments within the growing neurites (Fig. 2L), and to a lesser extent, IKAP is present at the soma region (Fig. 2K, indicated by an arrow). Interestingly, this vesicular pattern of IKAP localization is similar to one that we previously described in PNS neurons derived from human embryonic stem cells [23].

### *Ikbkap* downregulation does not affect NCC migration

It was previously described that IKAP localizes to membrane ruffles and regulates cell migration in several cell types [24], but this molecular mechanism was not yet tested in migrating NCC. Here we show that in migrating NCC IKAP colocalizes with distinct Tuj1 rich structures at the leading edges of lamellipodia (Fig. S1B–D, white arrow), and in *ikbkap* siRNA treated NCC, such structures are disrupted (Fig. S1F–H, red arrow). However, despite this observed phenotype as consequence to *ikbkap* downregulation, no significant difference in NCC migration or neuronal numbers was detected either *in vitro* or *in vivo* (Fig. S1I–N). These results confirm a previous report by Hunnicutt and colleagues [17], and George and colleagues [16], which arrived to a similar conclusion that *ikbkap* downregulation does not affect NCC migration, pathfinding and DRG formation.

### IKAP is involved in neurite outgrowth and peripheral target innervation *in vivo*

**Figure 3:**
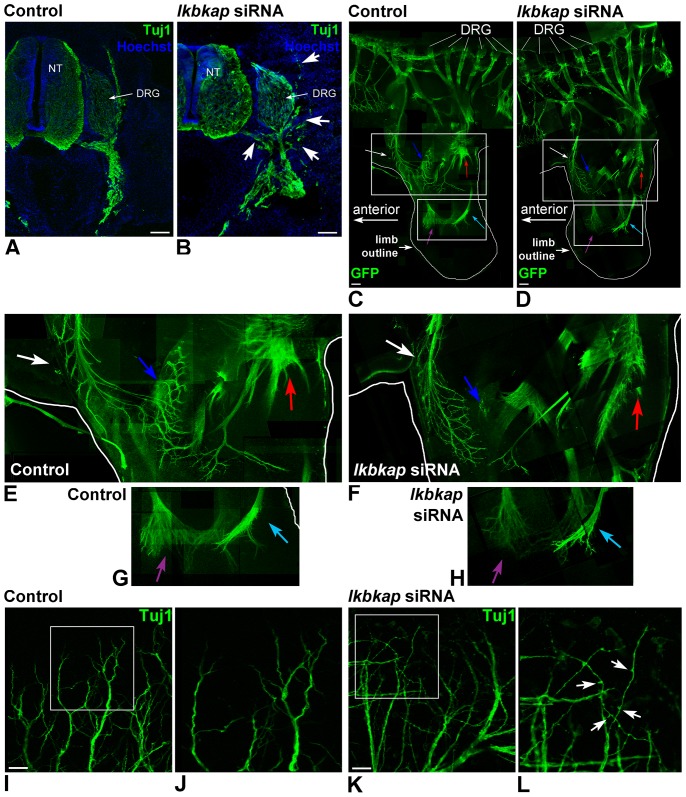
*Ikbkap* downregulation affects target innervation *in vivo*. (**A–B**) The embryos were electroporated with control or *ikbkap* specific siRNA at E2/HH11, and allowed to develop until E6. The transverse serial sections were stained with Tuj1 antibody to display neuronal patterns and with Hoechst 33342 to visualize nuclei. In *ikbkap* downregulated embryos abnormal peripheral nerve projections are visualized at various positions (B, white arrows) compared to control embryos (A), n = 5 embryos per treatment. Size bar 100 µm. (**C–H**) To visualize growing nerves, the embryos were co-electroporated with control siRNA plus pCAAG GFP expressing vector or *ikbkap* specific siRNA plus pCAAG GFP expressing vector at E2/HH11, and allowed to develop until E6 (N = 6). Representative tiling reconstruction with serial z-planes composed of multiple images of GFP labeled nerves taken at the lumbar and hindlimb regions (outlined in white) of control siRNA treated embryos are shown in (C, E, G), and of *ikbkap* specific siRNA treated embryos in (D, F, H). Boxed areas in C and D are magnified in E–G and F–H respectively. Size bar 100 µm. (**I–L**) Close up of skin innervations in abdomen region of Tuj1 stained whole mount embryos from previous experiment. I, J – control siRNA treated embryos; K, L – *ikbkap* specific siRNA treated embryos. White arrows indicate abnormal branching points. Size bar 10 µm.

To test the IKAP role at the stages of neural outgrowth, the embryos were electroporated either with control or *ikbkap* specific siRNA at E2/HH11, and allowed to develop until E6. The transverse serial sections from these embryos were stained with Tuj1 antibody to display neuronal patterns (Fig. 3A–B). We found that in *ikbkap* downregulated embryos, the peripheral projections of the DRG neurons were markedly disturbed and axons were misguided at the ventral root exit from the spinal cord (Fig. 3B, white arrows), while in the control embryos the exit routes from the spinal cord and DRG were well defined (Fig. 3A). These neuronal guidance abnormalities were further confirmed in whole mount E6 embryo preparations. For these experiments, the embryos were electroporated at E2/HH11 with control siRNA or with *ikbkap* specific siRNA, supplemented with pCAAG GFP expressing plasmid to visualize the electroporared targets. The embryos showing strong GFP fluorescence were allowed to grow until stage E6. Figure 3C–D shows representative tiling reconstruction with serial z-planes composed of multiple images of GFP labeled nerves taken at the lumbar and hindlimb regions (outlined in white) in control (Fig. 3C), and *ikbkap* specific siRNA treated embryos (Fig. 3D), (n = 6 per group). Abnormal growth of lumbar nerves innervating the hind limb was observed in *ikbkap* downregulated compared to control embryos (see colored arrows). Higher magnification images in boxed regions at the proximal hind limb position (Fig. 3E–F) show anterior branches (labeled by white arrow), midline branches (labeled by blue arrow), and posterior branches (labeled by red arrow) and display diverse phenotypes between the control siRNA (Fig. 3E) and *ikbkap* siRNA (Fig. 3F) treatments. At the anterior position (white arrow), we can observe that the nerve fibers in *ikbkap* siRNA treated embryos are more ramified bearing more branches at the axon terminals. In contrast, at the medial region of the limb (blue arrow) a whole nerve branch seems to be absent in *ikbkap* siRNA (Fig. 3F) in comparison with control siRNA treated embryos (Fig. 3E). The red arrow indicates the site where a nerve branch shows fewer ramifications in *ikbkap* siRNA in comparison to the same branch at the same position in the control. Figure 3G and H show magnification of boxed areas at the most distal outgrowing nerve ends in hind limbs of control siRNA and *ikbkap* siRNA treated embryos respectively. The growing nerves innervate the hindlimb in a similar manner, but the axonal ends in *ikbkap* siRNA treated embryos seem to be less developed (Fig. 3G, light blue and purple arrows). Similar confocal microscopy analysis was performed in the dermis of the abdomen stained with Tuj1 antibodies to visualize the PNS network. Misguided and aberrant branching is clearly observed in the *ikbkap* downregulated neurons (Fig. 3K and L) compared to the control (Fig. 3I and J). Note the multiple emerging branching points observed in *ikbkap* downregulated axons (see arrows Fig. 3L). In conclusion, these results indicate that IKAP is involved in fine tuning of the innervation process involving branching and positioning of small processes, while the positioning and lengths of the main nerves seems to be unaffected by *ikbkap* downregulation. Supporting these findings, we show in DRG dissociated cultures that *ikbkap* downregulation affect neuronal network formation, increasing adhesion between cells and branching (Fig. S2).

### *Ikbkap* downregulation affects tubulin structure in growth cones

**Figure 4:**
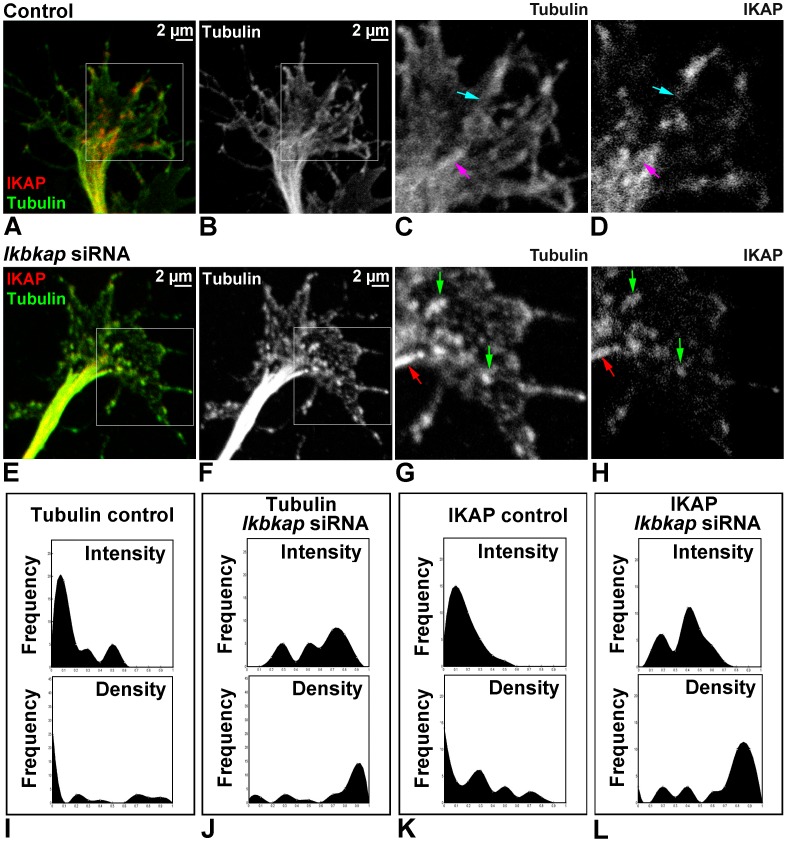
*Ikbkap* downregulation affect β-tubulin structure in growth cone. DRG from lumbar region of E10 embryos were electroporated with control or *ikbkap* specific siRNA, grown on laminin for 48 hours as described in methods, and stained with IKAP (red) and β-tubulin (green) antibodies. (**A–H**) Representative confocal images of the growth cone areas of control (A–D) and *ikbkap* siRNA treated (E–H) neurons. Boxed areas in A–B and E–F are magnified in C–D and G–H respectively. Colored arrows indicate IKAP localization along stable tubulin fibers (magenta arrows in C and D; red arrows in G and H), and along dynamic tubulin fibers (light blue arrows in C and D; green arrows in G and H). (**I–L**) Histograms representing the fluorescence intensities and densities of IKAP and β-tubulin signals at the growth cone area measured from multiple images by custom image analysis tool (see Methods).

We further aimed to investigate whether previously observed IKAP-dependent disturbances in target innervations can be explained by deregulation of the cytoskeleton. We demonstrate that in growth cones of control DRG cultures (Fig. 4A–D) IKAP is localized mostly along stable tubulin fibers (magenta arrows), and to less extent along dynamic tubulin fibers (light blue arrows) in filopodia and lamellipodia. In IKAP depleted growth cones (Fig. 4E–H), normal tubulin structures are disturbed and dynamic tubulin fibers seems to be fragmented and partly co-localized with IKAP (green arrows). To characterize changes in IKAP and tubulin localization in the growth cones, we compared IKAP and tubulin intensity and density distributions in a set of pictures from three independent experiments (Fig. 4I–L) using a custom algorithm as described in methods. In control growth cones (Fig. 4I and 4K), we observe large numbers of low intensity IKAP and tubulin pixels, whereas in *ikbkap* downregulated cultures (Fig. 4J and 4L) we observe low numbers of high intensity pixels. The same dynamics were observed in density analysis, confirming aggregation of IKAP and tubulin in *ikbkap* downregulated growth cones.

### *Ikbkap* downregulation affects JNK and NGF signaling in DRG neurons

**Figure 5:**
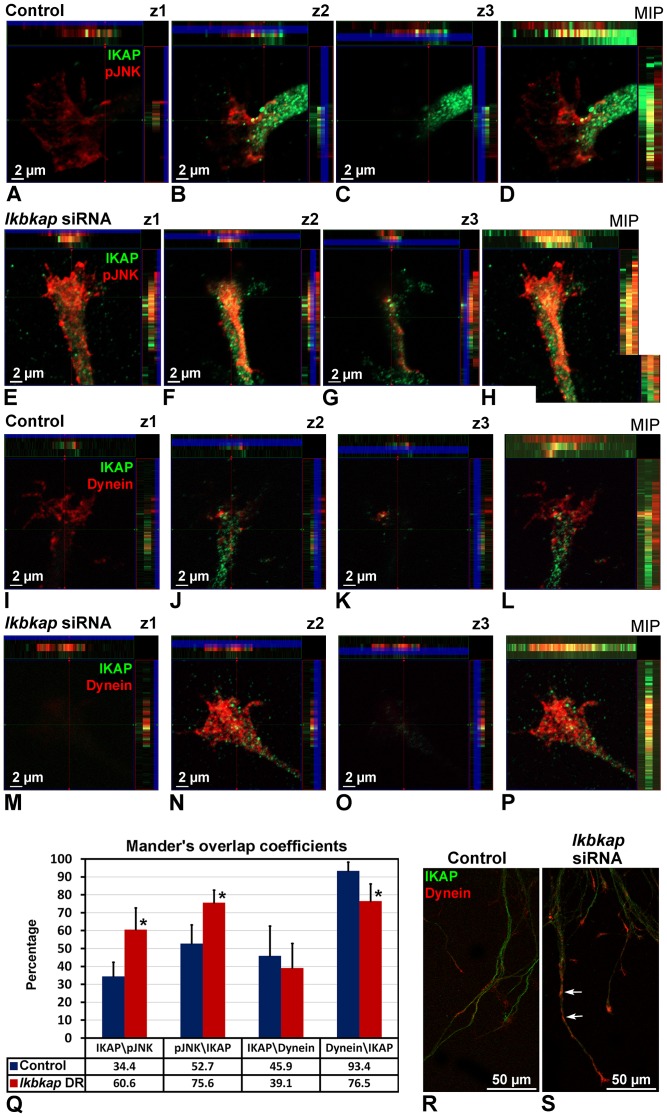
*Ikbkap* downregulation affects pJNK and dynein localization in growth cones. DRG from lumbar region of E10 embryos were electroporated with control or *ikbkap* specific siRNA, grown on laminin for 48 hours as described in methods, and stained for IKAP, phosphorylated JNK (pJNK) (**A–H**), or dynein (**I–P, R, S**). Confocal analysis of three serial z-sections of the growth cone was performed in images from three independent experiments. The total depth of the image z-stacks is 2.66 µm, z1 represents 0–0.88 µm, z2 represents 0.88–1.77 µm, and z3 represents 1.77–2.66 µm slices. (**Q**) Mander's overlap coefficient of IKAP-pJNK and IKAP-dynein localization was performed using ImageJ colocalization plugin (JACoP) as described in methods. Data are presented as mean ±SD. (**R, S**) Dynein accumulation along the developing neurites is observed in *ikbkap* downregulated cultures (**S**, arrows) compared with control neurites (**R**).

**Figure 6:**
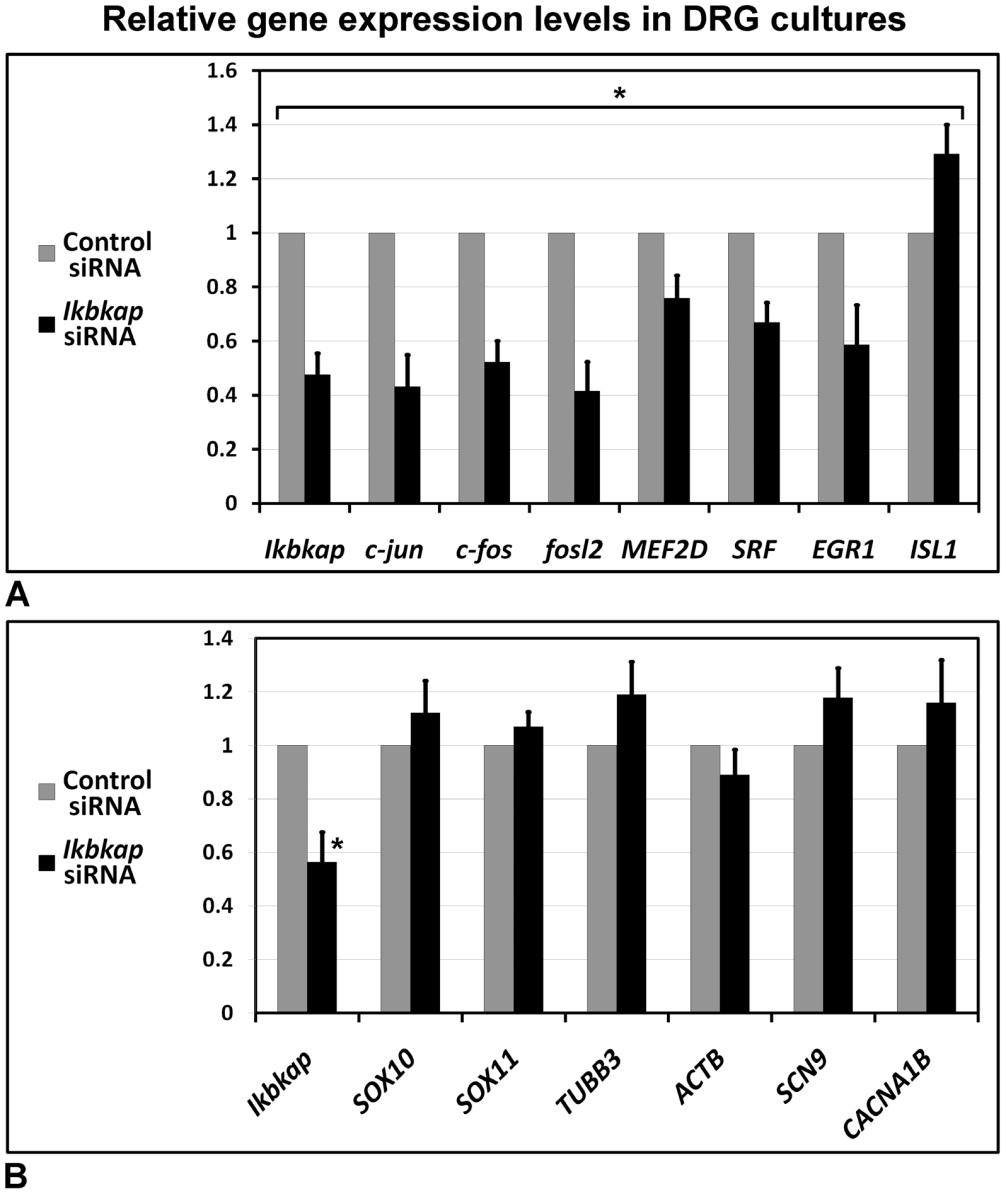
*Ikbkap* downregulation affects expression of pJNK and NGF responsive genes in DRG neurons. DRG from the lumbar region of E10 embryos were electroporated with control or *ikbkap* specific siRNA, grown on laminin for 48 hours, and processed for QRT-PCR as described in methods. Data are presented as relative gene expression levels of mean ±SD.

The results represented above show that *ikbkap* downregulation directly affects cytoskeletal morphology in axon terminals, which may have direct implications in microtubules dynamics, axonal growth, and axonal transport. Since IKAP is known to directly bind JNK [9], and JNK is known to regulate microtubules stability in neurons (reviewed in Sakakibara et al., [25]), we hypothesized that JNK functions might be affected in IKAP depleted neurons. In addition, we examined the possibility that IKAP is involved in retrograde transport by direct association with the motor protein dynein. To this end, we performed analysis of IKAP colocalization with active phosphorylated JNK (pJNK) and dynein in explanted DRG cultures taken from E10 embryos, which were electroporated with control or *ikbkap* specific siRNA and grown for 48 hours as described in methods (Fig. 5). Confocal analysis of a total depth of 2.66 µm divided in three fixed serial z-sections (of 0.88 µm z-step interval) at the growth cone area of axon terminals was performed in all images of three independent experiments. In control siRNA treated growth cones, specific JNK activation was mostly observed at the base z1 and middle z2 sections (Fig. 5A and B), but not at the top section z3 (Fig. 5C). p-JNK colocalization with IKAP mostly occurs at the z2 section (Fig. 5B and D). Similar colocalization pattern was detected for dynein and IKAP (Fig. 5I–L). In contrast, *ikbkap* siRNA treated growth cones show a spatial change in pJNK and dynein signals manifested by an increased ectopic pJNK aggregation colocalized with IKAP at all the 3 z-section levels (Fig. 5E–H), as well as aggregation of dynein with IKAP at the z-1 and z2 section levels (Fig. 5M–P). The colocalization between the signals was quantified using a colocalization image analysis tool (JACoP, ImageJ software) (Fig. 5Q) as described in methods. Interestingly, the results show that most of dynein and about a half of pJNK measured at the growth cone area in control culture conditions are associated with IKAP (93.4 and 52.7% respectively), but not vice versa for IKAP. As a result of *ikbkap* downregulation, a significant increase of about two folds in the proportion of IKAP which is colocalized with pJNK (IKAP/pJNK) was observed together with a significant increase (33%) in the proportion of pJNK which is colocalized with IKAP (pJNK/IKAP) at the growth cone area. The proportion of dynein co-localized with IKAP also appears to be affected (18% reduction) by *ikbkap* downregulation. Moreover, abnormal dynein accumulation along the developing neurites was observed in *ikbkap* downregulated cultures (Fig. 5S, white arrows) compared to control (Fig. 5R), suggesting a disturbance in dynein dependent transport in these cells. Overall, these results show that IKAP is spatially colocalized with pJNK and dynein and the localization pattern of expression in the axon terminus of both proteins is dependent of IKAP expression levels that may affect concomitantly axonal transport. To evaluate the *ikbkap* downregulation effect on potential dynein-dependent axonal transport disturbances and transcriptional regulation, we performed QRT-PCR analysis of known JNK responsive AP-1 genes *c-jun, c-fos*, and *fosl2*; along with known NGF induced immediate early transcription factors *MEF2D, SRF* and *EGR1* [26], [27], [28] in control and *ikbkap* downregulated DRG cultures (Fig. 6A). We observed significant reduction in the expression levels of the pJNK and NGF signaling dependent genes as a consequence of 54% reduction in *ikbkap* mRNA levels after 48 hours in culture. In parallel, we performed analysis of the expression of cytoskeletal genes Tuj1 and β-Actin, regulators of proliferation *SOX10* and *SOX11*, and calcium signaling genes *SCN9A* and *CACNA1B*, but their expression was not affected in respect to *ikbkap* downregulation (Fig. 6B). These results confirm our assumption that IKAP may be involved in transport of specific signals and demonstrate that *ikbkap* downregulation affects the expression of pJNK and NGF-inducible transcription factors in developing DRG neurons, establishing a mechanism that could explain the *in vivo* innervation phenotype described above (see Fig. 3 and 4), as well as increased neuronal cell death in FD neurons.

## Discussion

PNS development involves a well-coordinated sequence of events starting with NCC migration, differentiation, and target field innervations, leading to the establishment of properly sized and positioned peripheral neural network depending on specific environmental queues. A number of studies in different mouse conditional *ikbkap* mutant models were recently conducted to elucidate

neuronal depletion in the FD phenotype [13], [16], [29]. Despite the consensus that IKAP ablation does not affect NCC migration, neither DRG and SG formation, nor neuronal numbers until E12 stage in these mouse models, at later stages the results remains controversial. George and colleagues [16] found that IKAP-dependent TrkA (but not TrkC) sensory neuronal loss occurs at E12.5 due to Caspase-3 mediated apoptosis. In addition, premature cell cycle exit, differentiation, and cell death of Pax3 positive neural progenitors were found at this stage. In contrast, Jackson and colleagues [29] suggested that the majority of sensory and sympathetic neuronal loss caused by the absence of IKAP occurs as a result of neuronal failure to properly innervate their peripheral targets and to obtain adequate target-derived trophic support and not as a consequence of early abnormalities occurring during neurogenesis or differentiation. Moreover, in this work Jackson and colleagues showed that transgenic lineage specific dopamine β-hydroxylase sympathetic neurons depleted of *ikbkap* reveal normal sympathetic lineage marker expression, but showed significant target tissue innervation abnormalities and neuronal death.

Here we characterize the IKAP expression pattern in PNS development in the chick embryo and demonstrate that IKAP plays a specific role in neurite outgrowth, positioning, and target field innervation. We found that IKAP is specifically upregulated within growing axonal projections in postmitotic differentiated DRG neurons at the stages of peripheral target innervation (Fig. 1 and 2) and it is transported along growing neurites in vesicular-like structures. Moreover, we show that increase in inherent *ikbkap* expression in E6–E7 DRG coincides with the onset of peripheral outgrowth and target innervation (Fig. 1). *Ikbkap* downregulation at these stages affected axonal outgrowth and guidance leading to abnormal axonal branching and positioning of the axons innervating the developing hind limb (Fig. 3C–H), as well as abnormal innervation of the skin in the abdomen (Fig. 3I–L). These observations were also confirmed in dissociated DRG cultures demonstrating *ikbkap* downregulation effects on neurite branching and spatial network organization (Fig. S2). Interestingly, the majority of disturbances observed in peripheral innervation in these experiments occurred in branching and positioning of distal processes, especially in innervation of the skin, while the positioning of the main nerves seems to be unaffected. This suggests that IKAP may play a role in fine-tuning of the innervation process and the reading of environmental queues. In this respect we show that *ikbkap* downregulation directly affects growth cone morphology (Fig. 4) and probably axonal transport dependent signal transduction (Fig. 5 and 6). We show that IKAP protein is colocalized with tubulin, dynein, and pJNK in the growth cones, while *ikbkap* downregulation leads to tubulin aggregation as can be judged by shifts in the fluorescence intensity and density patterns (Fig. 4). These results indicate that IKAP is directly involved in tubulin organization in the growth cone, probably via its association with pJNK. Although IKAP ability to bind and regulate JNK activation in HEK-293 cells was described over a decade ago [9], here we show, for the first time, IKAP colocalization with pJNK at the growth cones in PNS neurons. This supports the view that IKAP could serve as a scaffold protein to facilitate known JNK dependent neuronal functions, such as axonal microtubule stabilization via phosphorylation of MAP1B, and microtubule plasticity via phosphorylation of superior cervical ganglion 10 protein (SCG10) in the growth cone (for review, Coffey, [30]). Interestingly, it was previously found that SCG10 is upregulated in FD cerebellum and fibroblasts [31], and in our *ikbkap* downregulated neuroblastoma model [32], possibly as part of the compensatory response to microtubule instability. In addition, JNK was found to phosphorylate kinesin 1 motor domain, which leads to dissociation of kinesin 1 from microtubules, regulating axonal transport [33]. Altogether, these observations support the view that IKAP is playing an essential role in the dynamics of microtubule reorganization and integration of signaling cues required for regulation of axonal outgrowth, branching, and gene expression in neurons. Concurrently, in this respect, we show for the first time that IKAP may be associated with dynein localization and may regulate in some way pJNK and NGF retrograde signaling in outgrowing neurons. We suggest that IKAP can play a role as a scaffold transport protein integrating extracellular signals (eg; neurotrophins like NGF or other) by selective binding to specific signaling cargoes (eg. pJNK) on one side and affinity binding to polymerizing microtubules in the growth cone at the other side. These diverse activities performed by IKAP allow specific transport of cargoes via dynein to the nucleus, regulating, in turn, the transcription of target genes, which contribute to many aspects of DRG neuronal function, including axon outgrowth and guidance, synapse maintenance, and cell survival. Supporting this hypothesis, we previously found in neuroblastoma gene expression microarray that IKAP deficiency induced the expression of several cytoskeleton and actin-binding proteins, while mostly, genes of axon guidance, axonal growth, and synapse structure and function where significantly downregulated [34], supporting IKAP role in neuronal outgrowth, guidance, and function. Specifically, axon guidance genes like Semaphorins, DPYSL3 (Dihydropyrimidinase necessary for Sema3 signaling), Ret and other transmembrane receptor protein tyrosine kinases, NetrinG1 and other extracellular matrix proteins, TNR (tenascinR), TNC (tenascinC), and NAV2 (neuron navigator) were affected. Thus, in turn, the outgrowth and branching phenotypes observed here in *ikbkap* downregulated DRG neurons in the developing chick embryo can be explained by the IKAP effects on multiple gene targets involved in these processes.

Altogether, the innervation role and the suggested involvement of IKAP in intracellular target derived signal transduction, specific gene expression together with cytoskeleton regulation in PNS neurons may explain in many ways the complexity of the FD phenotype that involves the selective loss of certain PNS neurons during development and after birth in FD patients [35], [2].

## Materials and Methods

### siRNA treatment

The embryos were staged according to Hamburger and Hamilton [36]. Four chick *ikbkap* specific or control scramble siRNA duplexes (with or without Cy3 label) were designed and purchased at Sigma Genosys (Table S1). *Ikbkap* specific siRNA duplexes were used in mix or interchangeably in different experiments. The siRNAs (0.5 µg/µl final concentration) in Fast Green dye solution (Sigma) were microinjected into the neural tube lumen at stage E2/HH11 embryos, and electroporated (BTX830 system, Molecular Delivery Systems) with square pulse (8 pulses of 25 mV for 50 msec with a 1 sec interval) using homemade tungsten electrodes placed at the two sides of the embryo. The efficiency of electroporation was assessed by siRNA labeled Cy3 fluorescence, and efficiency of *ikbkap* downregulation was evaluated by quantitative real-time PCR (QRT-PCR). Electroporation of dissected DRG was performed similarly in 24 well dish plates in 1 ml of fresh egg albumin, to reduce siRNA leakage.

### Neural tube explants

After electroporation, the embryos at the 13 somite stage were selected. Neural tubes were excised in 2% pancreatin solution (Sigma) at the defined trunk region (Fig. S1I, red rectangle). The neural tubes were grown on fibronectin coated (15 µg/ml, Sigma) coverslips in DMEM supplemented with 10% FBS, 2 mM Glutamax, 1 mM sodium pyruvate, 1% of *non-essential amino acid* stock, 100 µg/ml penicillin, and 100 mg/ml streptomycin.

### DRG cultures

Electroporated whole DRG or dissociated single cell neural progenitors (obtained from DRG with gentle trituration in 0.25% trypsin solution) were grown on laminin (5 µg/ml) coated coverslips in neurobasal medium (Invitrogen), supplemented with 2% B27 supplement (Invitrogen), 2 mM Glutamax (Invitrogen), 1 mM sodium pyruvate, 1% of *non-essential amino acid* stock, 100 µg/ml mg/ml penicillin, 100 mg/ml streptomycin, and 20 ng/ml rhNGF (R&D systems).

### Immunofluorescence of cells, embryos, and frozen sections

The embryos or cell cultures were fixed with 4% PFA, washed thoroughly with PBT (PBS/0.01%Triton X-100), permeabilized with PBS/1% Triton X-100, blocked with PBS/2%BSA/10%FBS/0.05%Triton X-100 for 1 hour, and incubated with appropriate primary antibodies in blocking solution at 4°C overnight (whole mount embryos were incubated at 4°C for 48 hours with agitation). The specimens were washed several times with PBT and incubated with appropriate secondary antibodies (for 3–6 hour with agitation for whole mount embryos; 1 hour for cultures). Hoechst 33342 dye (0.02 mg/ml) was added to the secondary antibody solution for nuclear staining. The specimens were rinsed and prepared for visualization. For staining in frozen section, the embryos were snap frozen in OCT-embedding medium. Transverse sections of the embryos (16 µm thick), were immediately fixed with ice cold methanol for 5 minutes, washed in PBS, and stained as described above. Antibody sources are listed in Table S1.

### Quantitative Real Time PCR

RNA from at least 3 biological replicates was extracted using RNeasy Kit (Qiagen) and reverse transcribed with Applied Biosystem Kit. QRT-PCR was performed in technical triplicates using Cyber green mix (AB gene, Surrey, UK) and Rotor-Gene 6000 (Qiagen) workstation. *HPRT1* expression was taken to normalize gene expression levels. Primer sequences are listed in Table S1.

### Image analysis

For IKAP fluorescence levels quantification (see Fig. 2I), CTCF (Corrected Total Cell Fluorescence) was calculated using ImageJ software as follows:

CTCF  =  Integrated Density - (Area of selected cell X Mean fluorescence of background readings).

Briefly, NCC cell borders were selected using actin staining as shown in Fig. 2F, upper panel), Neuron cell borders were selected using Tuj1 staining as shown in Fig. 2F, middle panel. These cell borders selections were overlaid on the IKAP stained pictures (Fig. 2F, bottom panel). Then to calculate CTCF the Area, Integrated Density, and Mean Gray Value as well as the background measurements of IKAP expression were measured in NCC cells and neurons using ImageJ, In the regions where neurons are located above the NCC, average NCC CTCF was substracted from total CTCF value to obtain neuronal CTCF value. Sample number N = 15 neurons versus 15 NCC.

For measuring IKAP and Tubulin expression levels in Fig. 4, custom MATLAB scripts were used to test intensity and density of the protein expression in the growth cones. Intensity values were defined as (Sum of intensities in user defined polygon area)/(Polygon area). Density values were defined as (Number of pixels above density threshold in user defined polygon area)/(Polygon area).

For measuring IKAP, pJNK and Dynein expression levels in Fig. 5, confocal images with resolution 1024×1024 pixels were used for analysis. The expression pattern of the stained proteins was assessed by making maximum intensity projections. For colocalization analysis, we used ImageJ Subtract Background function followed by Intensity Correlation Analysis (JaCoP plug-in) to obtain the Pearson's Correlation coefficient and Manders overlap coefficient (Bolte and Cordelieres, [37]).

### Statistical analysis

Data are presented as means ±SD. Statistical significance was analyzed using one-way ANOVA and Post hoc analysis using Tukey follow up test. The differences with p≤0.05 were referred as significant.

### Supporting information

Supporting information include Fig. S1, which describes experiments of *ikbkap* downregulation in migrating NCC and in early DRG *in vitro* and *in vivo*; Fig. S2, showing *Ikbkap* downregulation effect on the network formation in DRG dissociated cultures; Table S1, containing the list of siRNA and the primer sequences, and Table S2, containing list of antibodies used in this study.

### Ethics Statement

In all our experiments, the embryos were euthanized by decapitation starting stage E4. According to the local guidelines, experiments with chick embryos at stages used in this study (between E2 and E18) do not require IACUC approval. The Tel Aviv IACUC is working according to the following rules: The state law of prevention of animal cruelty (animal experimentations) 1994; Guidelines for preventions of animal cruelty (animal experimentations) 2001 published by the council for animal experimentation and The NRC Guide for the Care and Use of Laboratory Animals.

## Supporting Information

Figure S1 **IKAP does not affect NCC migration** ***in vivo*** **and** ***in vitro*** **.** The embryos were electroporated at E2/HH11 with control or *ikbkap* specific siRNA. The neural tubes containing premigratory NCC were excised from specific trunk region (I, red rectangle) and grown in fibronectin coated dishes allowing NCC migration as explained in methods. (**A-H**) Confocal micrographs of IKAP localization in migrating NCC (24 hours in culture). Figures B-D and F-H show high magnification of the outlined regions in A and E, respectively. IKAP show a vesicular localization, partially colocalized with Tuj1 in the cell body, and also abundant in Tuj1 rich distinct structures at the leading edges of lamellipodia (B-D, white arrow). In IKAP downregulated NCC, such structures are disrupted (F-H, red arrow). (**J**) After 48 hours in culture, the numbers of migrated cells were counted for each explants (n = 15 explants analyzed per treatment). (**K-N**) The embryos were electroporated at E2/HH11 with control or *ikbkap* specific siRNA, *in ovo* and returned to the incubator. (**K-L**) After 24 hours, the embryos were excised from the egg, fixed, and stained with HNK-1 antibodies. Migrating HNK-1 positive NCC were counted in 15 µm thick trunk transverse sections of 300 µm of total length (A total of 20 sections were counted per embryo and 5 embryos were analyzed per treatment). (**M**) After 4 days of incubation, the embryos which reach E6 stage were excised from the egg, fixed, and migration distance (MD) was evaluated in transverse sections (the distance from the dorsal margin of the DRG to the dorsal margin of the spinal cord). (**N**) Quantification was performed in three central serial sections per DRG, three DRGs per embryo were examined, and five embryos were included per treatment. Data are presented as mean ±SD.
(TIF)

Figure S2 ***Ikbkap*** **downregulation affect network formation in DRG dissociated cultures.** DRG from lumbar region of E10 embryos were electroporated with control or *ikbkap* specific siRNA, dissociated to single cells, and plated on laminin at density 100,000 cells/well at 24 well plate as described in methods. After 8 days *in vitro*, neural networks were formed. The cultures were fixed, stained with Tuj1 antibodies (green) and Hoechst 33342 (blue) to visualize nuclei, and high resolution images were obtained using IN Cell Analyzer 1000 (GE healthcare). N = 6 repeats for treatment, 3 independent experiments. (**A–B**) Representative images of neuronal networks. (**C–D**) For quantitative analyses, the neurites stained by Tuj1 were outlined free handed at high magnification (red), so that the neuronal network from images were faithfully reconstructed. (**E**) Efficiency of *ikbkap* downregulation was evaluated by QRT-PCR after 72 h in culture and showed a 65% reduction in *ikbkap* mRNA levels. *Ikbkap* siRNA treated neurons form larger cell clusters than control siRNA treated neurons, resulting in a significantly lower number of single cells measured per field (**F**). Individual neurites in *ikbkap* downregulated cultures seem to be thinner with a higher number of branching points per field than those neurites in the control culture (**G**). Size bar 50 µm. Data are presented as mean ±SD.
(TIF)

Table S1 **List of siRNA and primer sequences.**
(DOCX)

Table S2 **List of antibodies.**
(DOCX)
