# Supplementary figures and images for: Involvement of IKAP in Peripheral Target Innervation and in Specific JNK and NGF Signaling in Developing PNS Neurons
Source: PLoS One. 2014 Nov 19;9(11):e113428. doi: 10.1371/journal.pone.0113428 (PMC4237409; doi:10.1371/journal.pone.0113428)

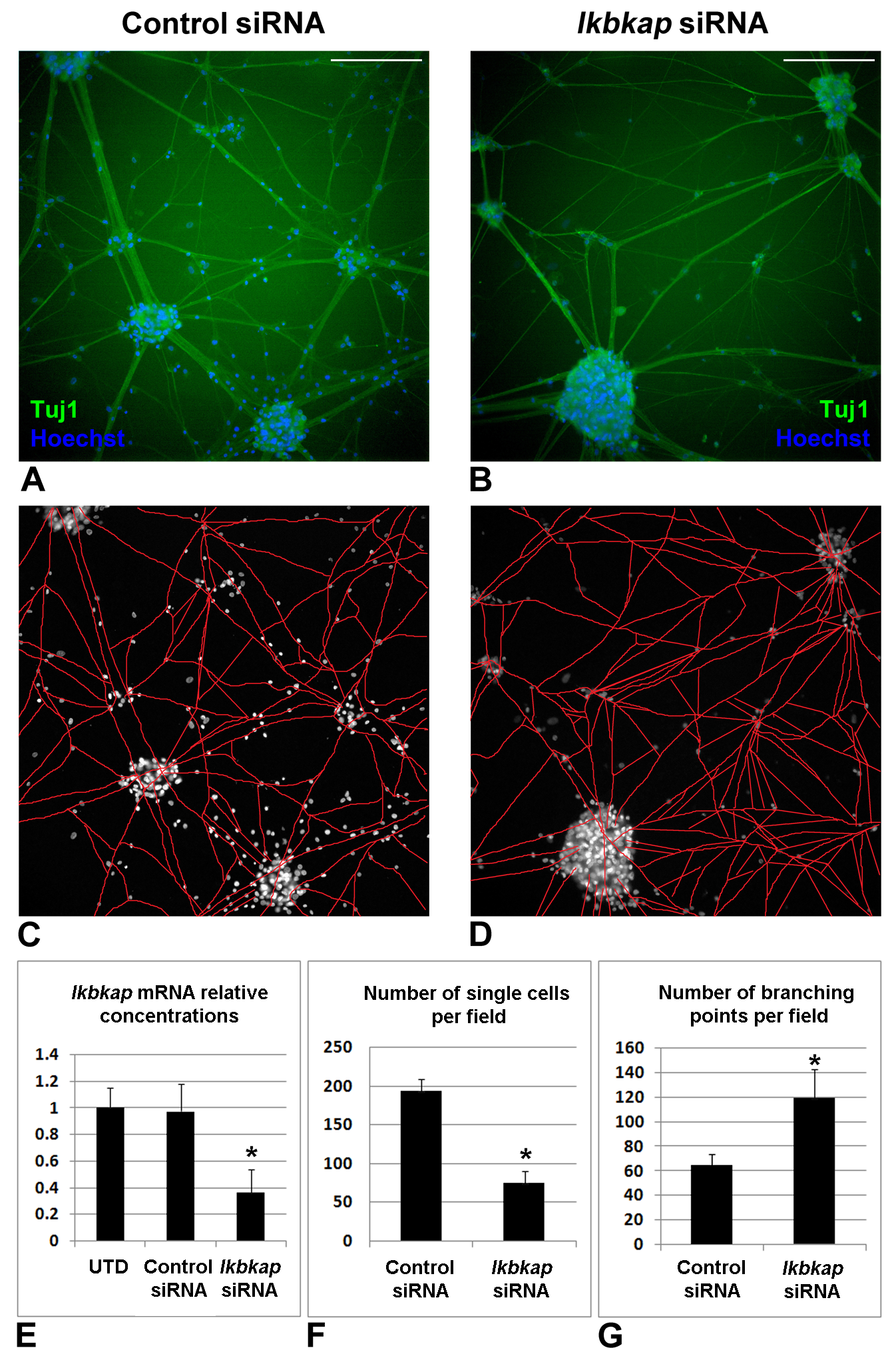

Supplement: Figure S2 — Ikbkap downregulation affect network formation in DRG dissociated cultures. DRG from lumbar region of E10 embryos were electroporated with control or ikbkap specific siRNA, dissociated to single cells, and plated on laminin at density 100,000 cells/well at 24 well plate as described in methods. After 8 days in vitro, neural networks were formed. The cultures were fixed, stained with Tuj1 antibodies (green) and Hoechst 33342 (blue) to visualize nuclei, and high resolution images were obtained using IN Cell Analyzer 1000 (GE healthcare). N = 6 repeats for treatment, 3 independent experiments. (A–B) Representative images of neuronal networks. (C–D) For quantitative analyses, the neurites stained by Tuj1 were outlined free handed at high magnification (red), so that the neuronal network from images were faithfully reconstructed. (E) Efficiency of ikbkap downregulation was evaluated by QRT-PCR after 72 h in culture and showed a 65% reduction in ikbkap mRNA levels. Ikbkap siRNA treated neurons form larger cell clusters than control siRNA treated neurons, resulting in a significantly lower number of single cells measured per field (F). Individual neurites in ikbkap downregulated cultures seem to be thinner with a higher number of branching points per field than those neurites in the control culture (G). Size bar 50 µm. Data are presented as mean ±SD. (TIF) [file pone.0113428.s002.tif]
