# Supplementary material for: Involvement of IKAP in Peripheral Target Innervation and in Specific JNK and NGF Signaling in Developing PNS Neurons
Source: PLoS One. 2014 Nov 19;9(11):e113428. doi: 10.1371/journal.pone.0113428 (PMC4237409; doi:10.1371/journal.pone.0113428)
Supplement: Table S1 — List of siRNA and primer sequences. (DOCX) [file pone.0113428.s003.docx]

**Table S1. List of siRNA and primer sequences**

| **siRNA sequences** | |
| --- | --- |
| Chick *ikbkap* 1 (targets 337-356 bp) | GAGACAUCCUUCUUUGCAAdTdT |
| Chick *ikbkap* 2 (targets 1247-1266 bp) | CAUUGAUGGAGCUAAAGUAdTdT |
| Chick *ikbkap* 3 (targets 1703-1722 bp) | GGAAGAAGUUCUGCAUGUUdTdT |
| Chick *ikbkap* 4 (targets 1990-2009 bp) | UCACUUCGUUUGCAACAUAdT |
| eGFP | GCAAGCUGACCCUGAAGUUCAUdTdT |
| **Primer sequences** | |
| *HPRT1* fwd | CTGTGTACTCAAGGGTGGCT |
| *HPRT1* rev | TCCACAGTCATGGGGATTGAC |
| *ikbap* (IKAP) fwd | GTTGGTCAGGATCAGCAAGC |
| *ikbap* (IKAP) rev | ACCGTAAACTGAGGTGCTCT |
| *ISL1* (Islet-1) fwd | GACTTCGCATTGCAGAGTGAC |
| ISL1 (Islet-1) rev | GATGCCACTTCACTTCCGGT |
| *RARB* (RAR-β) fwd | CTCACCAGGAAACCTTCCCC |
| *RARB* (RAR-β) rev | ATTTGTCCCAGAGCCCAAGG |
| *CACNA1B* fwd | ACTCCGTAGGCAACAGCAAA |
| *CACNA1B* rev | GTTCTTGTCTTCCTCGGCCA |
| *SCN9A* fwd | TGCTGATGAAAAGTCTAACCGTG |
| *SCN9A* rev | GTCTTTCCTGCCTCCAAGTCA |
| *ACTB* (β-Actin) fwd | ATTCTGTCTGGATTGGTGGCT |
| *ACTB* (β-Actin) rev | GATGGACTATGGAGGGGCCA |
| *TUBB3* (Tuj1) fwd | GAGCATGGCATAGACCCCAG |
| *TUBB3* (Tuj1) rev | CGAGGCACGTACTTGTGAGA |
| *MEF2D* fwd | AGCTCGTTGGTGACCCAATC |
| *MEF2D* rev | TGGGGACACCGTATTCCTCT |
| *SRF* fwd | TCATCCAGACGTGCCTCAAC |
| *SRF* rev | GTAGGTGAGGTCGGTCTCCT |
| *c-jun* fwd | GCATTGTTTGTGCTGCGTTC |
| *c-jun* rev | GCTCTTTTTGCGTGGGTTTGA |
| *c-fos* fwd | ATGATGTACCAGGGCTTCGC |
| *c-fos* rev | GACGGGTAGTAGGTGAGGCT |
| *fosl2* fwd | GCCATCACTCCTGGTTCCTC |
| fosl2 rev | GTGAGCTTTGGAGCAGGACT |
| *EGR1* fwd | CTTGCGGCAGACACTTTTCC |
| *EGR1* rev | TAATTGGCGGTAGCCGTGTT |
| *SOX10* fwd | GCAGCCTTCACAGGGTTTG |
| *SOX10* rev | CATCTCCACCTCCGACAGAT |
| *SOX11* fwd | CAGCGAGAAGATCCCCTTCA |
| *SOX11* rev | TTTTGGGCTTTTTCCTGGGC |
