# Supplementary material for: Involvement of IKAP in Peripheral Target Innervation and in Specific JNK and NGF Signaling in Developing PNS Neurons
Source: PLoS One. 2014 Nov 19;9(11):e113428. doi: 10.1371/journal.pone.0113428 (PMC4237409; doi:10.1371/journal.pone.0113428)
Supplement: Table S2 — List of antibodies. (DOCX) [file pone.0113428.s004.docx]

**Table S2. List of antibodies**

| **Antibody description** | **Supplier** | **Final dilution** |
| --- | --- | --- |
| Anti IKAP rabbit polyclonal IgG | Santa Cruz, H-302 | 1:500 |
| Anti Tuj1 (neuronal class III beta tubulin) mouse igG | Covance, MMS-435P | 1:1000 |
| Anti HNK-1 mouse IgG | DSHB, 1C10 | 1:200 |
| Anti Islet-1 mouse IgG | DSHB, 39.4D5 | 1:200 |
| Anti β-tubulin mouse IgG | Sigma, AA2 | 1:1000 |
| Anti pJNK rabbit IgG | R&D systems, AF-1205 | 1:500 |
| Anti pJNK mouse IgG | Santa Cruz, SC-6254 | 1:500 |
| Anti dynein mouse IgG | Gift from Eran Perlson | 1:1000 |
| Phalloidin-TRITC | Sigma | 1:7000 |
| Secondary antibodies (Alexa 488, 555, and 647 conjugated) | Abcam | 1:1000 |
